# Diagnostic Magnetic Resonance Imaging of Atherosclerosis in Apolipoprotein E Knockout Mouse Model Using Macrophage-Targeted Gadolinium-Containing Synthetic Lipopeptide Nanoparticles

**DOI:** 10.1371/journal.pone.0143453

**Published:** 2015-11-16

**Authors:** Zu T. Shen, Shaokuan Zheng, Matthew J. Gounis, Alexander B. Sigalov

**Affiliations:** 1 SignaBlok, Inc, Shrewsbury, Massachusetts, United States of America; 2 Department of Radiology, University of Massachusetts Medical School, Worcester, Massachusetts, United States of America; Katholieke Universiteit Leuven, BELGIUM

## Abstract

Cardiovascular disease is the leading cause of death in Western cultures. The vast majority of cardiovascular events, including stroke and myocardial infarction, result from the rupture of vulnerable atherosclerotic plaques, which are characterized by high and active macrophage content. Current imaging modalities including magnetic resonance imaging (MRI) aim to characterize anatomic and structural features of plaques rather than their content. Previously, we reported that macrophage-targeted delivery of gadolinium (Gd)-based contrast agent (GBCA-HDL) using high density lipoproteins (HDL)-like particles significantly enhances the detection of plaques in an apolipoprotein (apo) E knockout (KO) mouse model, with an atherosclerotic wall/muscle normalized enhancement ratio (NER) of 120% achieved. These particles are comprised of lipids and synthetic peptide fragments of the major protein of HDL, apo A-I, that contain a naturally occurring modification which targets the particles to macrophages. Targeted delivery minimizes the Gd dose and thus reduces the adverse effects of Gd. The aims of the current study were to test whether varying the GBCA-HDL particle shape and composition can further enhance atherosclerotic plaque MRI and control organ clearance of these agents. We show that the optimized GBCA-HDL particles are efficiently delivered intracellularly to and uptaken by both J774 macrophages *in vitro* and more importantly, by intraplaque macrophages *in vivo*, as evidenced by NER up to 160% and higher. This suggests high diagnostic power of our GBCA-HDL particles in the detection of vulnerable atherosclerotic plaques. Further, in contrast to discoidal, spherical GBCA-HDL exhibit hepatic clearance, which could further diminish adverse renal effects of Gd. Finally, activated macrophages are reliable indicators of any inflamed tissues and are implicated in other areas of unmet clinical need such as rheumatoid arthritis, sepsis and cancer, suggesting the expanded diagnostic and prognostic use of this method.

## Introduction

The buildup of plaque in arterial walls, better known as atherosclerosis, remains the leading cause of death in the United States and other Western cultures [1]. The large majority of cardiovascular events including stroke and myocardial infarction, result from the sudden rupture of vulnerable plaques in coronary arteries without any prior symptoms [2,3]. While both vulnerable and stable plaques can cause stenosis, only vulnerable plaques require clinical intervention as they are prone to rupture [4]. Thus, differentiating stable versus vulnerable plaques is of particular clinical interest. Currently, none of the commercially available diagnostic methods can detect vulnerable plaques before a cardiovascular event [5,6]. The overall goal of this method is to provide a diagnostic tool to detect vulnerable plaques and potentially offer life saving intervention.

Inflammation plays a key role in all stages of atherosclerosis with macrophages involved in plaque pathogenesis [7,8]. High and active intraplaque macrophage content correlates strongly with plaque vulnerability [9,10]. In coronary and carotid plaques, the number of lipid-laden macrophages has been found to be significantly higher in vulnerable plaques as compared to their stable counterparts, with a difference as high as 300–500% [10,11,12]. These findings suggest that macrophage content can be used as a distinctive marker for vulnerable plaques and macrophage-targeted imaging can be utilized to track plaque vulnerability.

Magnetic resonance imaging (MRI) is a powerful and noninvasive method for the imaging of atherosclerosis [13]. The use of contrast agents, including the gadolinium (Gd)-based contrast agents (GBCAs) in MRI greatly enhances the diagnostic power of MRI but still does not allow for the detection of macrophage-rich vulnerable plaques and the differentiation from stable plaques [13,14,15]. This suggests the need for a macrophage-specific vehicle for the targeted delivery of GBCAs to vulnerable plaques. In addition, the use of GBCAs has been associated with a serious condition known as nephrogenic systemic fibrosis (NSF), particularly in patients with impaired renal function [16,17]. Furthermore, a positive correlation between GBCA dose and increased side effects has been reported [18]. Therefore, the combined use of GBCA with a macrophage-targeted delivery system that could minimize GBCA dose and achieve hepatic clearance without compromising high diagnostic power would be a valuable tool for vulnerable plaque detection.

High-density lipoproteins (HDL) are a group of native lipoproteins whose primary role is to transport excess cholesterol from the peripheral tissues to the liver [19]. One of the advantages of using HDL as a delivery vehicle is that HDL particles can be readily reconstituted using lipids and apolipoproteins (apo) *in vitro* [20]. However, unmodified native HDL do not target macrophages, therefore additional targeting moieties are needed. A recent study demonstrated the use of the apo E-derived lipopeptide incorporated into GBCA-HDL, which was found to enhance the contrast of atherosclerotic plaques [21]. However, this cationic and detergent-like molecule has been found to exert neurotoxic effects and has been linked to the etiology of Alzheimer’s disease [22,23]. Another study utilized a monoclonal antibody against the macrophage scavenger receptor to target the GBCA-containing micelles to macrophages [24]. While contrast enhancement was also achieved in atherosclerotic plaques, the use of monoclonal antibodies as targeting moieties can often result in undesired side effects [25].

To avoid the pitfalls of targeting moieties, we have recently developed GBCA-HDL with apo A-I that contains a naturally occurring oxidative modification, which targets the particles to macrophages both *in vitro* and *in vivo* [26]. This significantly enhances the detection of macrophage-rich atherosclerotic plaques in the apo E knockout (KO) mouse model of atherosclerosis, with an atherosclerotic wall/muscle normalized enhancement ratio (NER) of up to 120% [26]. The rationale for utilizing this modification is derived from the fact that two of the three methionines (Met-112 and Met-148) in apo A-I are known to be susceptible to oxidation and both oxidized and unoxidized forms of apo A-I occur *in vivo* [27,28,29]. Importantly, oxidized apo A-I has been found in human aortic lesions and its content positively correlates with increased disease severity [30]. Furthermore, we have demonstrated that the ability for modified apo A-I to target GBCA-HDL to macrophages can be achieved using synthetic 22-mer peptides corresponding to apo A-I amphipathic helices 4 (H4) and 6 (H6), which contain methionines 112 and 148, respectively [26]. The use of synthetic peptides in place of native apo A-I isolated from human plasma avoids potential clinical and regulatory complications associated with using human protein. In this study, we increased the GBCA content per GBCA-HDL nanoparticle, which resulted in an atherosclerotic wall/muscle NER increase to greater than 160% in apo E KO mice at the same administration dose of Gd. Furthermore, our results indicate that hepatic clearance of GBCAs can be achieved by adjusting the shape and composition of the HDL particle, which should minimize the risks associated with NSF. The demonstrated diagnostic power of this method in detecting macrophage-rich atherosclerotic plaques combined with the desirable hepatic clearance of the contrast agents used strongly encourage its further development for identifying vulnerable patients.

## Materials and Methods

### Chemicals and Lipids

1,2-dimyristoyl-*sn*-glycero-3-phosphocholine (DMPC), 1,2-dimyristoyl-*sn*-glycero-3-phospho-(1′-rac-glycerol) (DMPG), 1-palmitoyl-2-oleoyl-*sn*-glycero-3-phosphocholine (POPC), 1,2-dimyristoyl-*sn*-glycero-3-phosphoethanolamine-N-diethylenetriaminepentaacetic acid (gadolinium salt) (14:0 PE-DTPA-Gd), 1,2-dimyristoyl-*sn*-glycero-3-phosphoethanolamine-N-(lissamine rhodamine B sulfonyl) (ammonium salt) (Rho B-PE) and cholesterol were all purchased from Avanti Polar Lipids (Alabaster, AL, USA). Cholesteryl oleate, sodium cholate and other chemicals were purchased from Sigma-Aldrich (St. Louis, MO, USA).

### Cells

The murine macrophage cell line J774A.1 (TIB-67) was obtained from the American Type Culture Collection (ATCC, Manassas, VA, USA).

### Peptides

Synthetic oxidized peptides H4 and H6 corresponding to apo A-I helices 4 and 6 were purchased from American Peptide Company (Sunnyvale, CA, USA).

### Dylight 488 Labeling of Oxidized Apo A-I H4 Peptide

Oxidized apo A-I H4 peptide was solubilized using 0.1M phosphate, pH 8 and reacted using a 2-fold molar excess of Dylight 488 N-Hydroxysuccinimide (NHS) ester (ThermoFisher Scientific, Waltham, MA, USA) and incubated at 25°C for 3 h. The reaction was quenched using a 25-fold molar excess of ethanolamine (Sigma-Aldrich, St. Louis, MO, USA) relative to the NHS ester. The reaction mixture was purified using the BioCAD 700E High Pressure Liquid Chromatography (HPLC) system (Applied Biosystems, Carlsbad, CA, USA) fitted with a reverse phase (RP) HPLC column (Grace Vydac, Hesperia, CA, USA).

### Preparation and Purification of Discoidal and Spherical Paramagnetic and Fluorescent Lipoproteins

The discoidal GBCA-HDL complexes were synthesized essentially as described [21,31,32] except no dialysis was undertaken. A molar ratio of 1:10:4:39:1, corresponding to oxidized apo A-I peptide:DMPC:DMPG:14:0 PE-DTPA-Gd:Rho B-PE was used. Briefly, DMPC, DMPG, 14:0 PE-DTPA-Gd and Rho B-PE in organic solvent(s) were combined and dried using a slow stream of nitrogen. The dried lipid films were placed under vacuum for 8 h. Lipid films were dispersed using phosphate-buffered saline (PBS), pH 7.4. After 30 min incubation at 30°C, oxidized apo A-I peptides H4 and H6 were added to the dispersed lipids. For confocal microscopy studies, Dylight 488-labeled oxidized apo A-1 peptide H4 was included. The reaction mixture was incubated for 3 h at 30°C and purified on a calibrated Superdex 200 HR gel filtration column (GE Healthcare Biosciences, Pittsburgh, PA, USA) using the BioCAD 700E Workstation. The purified discoidal GBCA-HDL particles were filter sterilized and stored at 4°C.

The spherical GBCA-HDL complexes were synthesized essentially as described [21,32,33]. A molar ratio of 1:3:1:60:103 corresponding to oxidized apo A-I peptide:cholesterol:cholesteryl oleate:lipid:sodium cholate was used, where the lipid component contained POPC:14:0 PE-DTPA-Gd:Rho B-PE in a corresponding molar ratio of 15:40:1. Briefly, POPC, cholesterol, cholesteryl oleate, 14:0 PE-DTPA-Gd and Rho B-PE in organic solvent(s) were combined and dried using a slow stream of nitrogen. The dried lipid films were placed under vacuum for 8 h. Lipid films were dispersed using Tris-buffered saline (TBS) containing 1 mM ethylenediaminetetraacetic acid (EDTA), pH 7.4. After 30 min incubation at 30°C, sodium cholate was added. The mixture was incubated for 30 min at 50°C in a water bath and oxidized apo A-I peptides H4 and H6 were added to the dispersed lipids. For confocal microscopy studies, Dylight 488-labeled oxidized apo A-I peptide H4 was included. The reaction mixture was incubated for 3 h at 30°C. After the incubation, the mixture was dialyzed 12 h at 25°C against PBS, pH 7.4 using Spectra/Por 7 dialysis membrane with 1000 molecular weight cut-off (Spectrum Laboratories, Rancho Dominguez, CA, USA). The dialyzed mixture was purified on a calibrated Superdex 200 HR gel filtration column using the BioCAD 700E Workstation. The purified spherical GBCA-HDL particles were filter sterilized and stored at 4°C.

### Macrophage Uptake of Paramagnetic and Fluorescent Lipoproteins *In Vitro* for Rhodamine B Fluorescence Analysis

Studies to quantify macrophage uptake of paramagnetic and fluorescent lipopeptide nanoparticles *in vitro* were performed as previously described [21,26]. Briefly, BALB/c murine macrophage J774A.1 cells were cultured at 37°C with 5% CO_2_ in Dulbecco’s Modification of Eagle’s Medium (DMEM, Cellgro, Mediatech Inc, Manassas, VA) with 2 mM glutamine, 100 U ml^-1^ penicillin, 0.1 mg ml^-1^ streptomycin and 10% fetal bovine serum (Cellgro, Mediatech Inc, Manassas, VA) and grown to approximately 90% confluency in 6 well tissue culture plates (Corning, Tewksbury, MA, USA). After reaching target confluency, cells were incubated for 2 h at 37°C using discoidal or spherical GBCA-HDL in complete DMEM at a concentration of 2 μM Rhodamine B. After incubation, the cells were washed twice using PBS and lysed using Promega Lysis Buffer. Rhodamine B fluorescence was measured on a Gemini XPS fluorescence microplate reader (Molecular Devices, Sunnyvale, CA, USA). The protein concentrations in the lysates were measured using Bradford Reagent (Sigma-Aldrich, St. Louis, MO, USA) and a MultiSkan Spectrum microplate reader (Thermo Fisher Scientific, Waltham, MA, USA) according to the manufacturer’s recommended protocol.

### Macrophage Uptake of Paramagnetic and Fluorescent Lipoproteins *In Vitro* for Confocal Analysis

To visualize macrophage uptake of paramagnetic and fluorescent lipopeptide nanoparticles *in vitro*, autoclaved 22 x 22 mm glass coverslips (VWR, Radnor, PA, USA) were placed into the bottom of 6 well tissue culture plates using ethanol-sterilized plastic forceps (VWR, Radnor, PA, USA). BALB/c murine macrophage J774A.1 cells were cultured at 37°C with 5% CO_2_ in DMEM with 2 mM glutamine, 100 U ml^-1^ penicillin, 0.1 mg ml^-1^ streptomycin and 10% fetal bovine serum and grown to approximately 50% confluency in 6 well tissue culture plates containing the glass coverslips. After reaching target confluency, cells were incubated for 2 h at 37°C using discoidal or spherical GBCA-HDL in complete DMEM at a concentration of 2 μM Rhodamine B. Cells were washed twice using Hanks Balanced Salt Solution (HBSS, GE Healthcare, Logan, UT, USA) and fixed using a 4% paraformaldehyde solution (Alfa Aesar, Heysham, Lancashire, UK) in HBSS for 50 min at 4°C. After the incubation, 50 μl of Prolong Gold antifade mounting medium with 4’,6-diamino-2-phenylindole (DAPI) (Invitrogen, Carlsbad, CA, USA) was added to the center of a standard glass microscope slide. The glass coverslip containing cells was transferred from the 6 well plate using tweezers, the edges were dabbed dry and the coverslip with the cells facing down was placed onto the mounting medium. The mountant was allowed to spread to the corners of the coverslip and any excess was removed. The mounted slide was allowed to cure at 25°C in the dark for at least 72 hrs. The mounted coverslip and slide was cleaned with water, followed by ethanol to remove any residual salt. Mounted slides were stored at 4°C and analyzed by confocal microscopy.

Confocal imaging was performed using a Leica TCS SP5 II laser scanning confocal microscope (Leica, Microsystems, Mannheim/Wetzlar, Germany) with acousto-optical beam splitter equipped with hybrid detectors, a detector for transmitted light including differential interference contrast (DIC) and eight laser lines at 405, 458, 476, 488, 496, 514, 561 and 633 nm. Leica Application Suite Advanced Fluorescence software was used to adjust laser and detector settings and to acquire data. Images were acquired using a 63x oil-immersion objective lens, hybrid detectors, a 405 nm laser for DAPI and DIC, a 488 nm laser for Dylight 488 and a 561 nm laser for Rhodamine B. Acquired images were analyzed using Leica Application Suite X.

### Macrophage Uptake of Paramagnetic and Fluorescent Lipoproteins *In Vitro* for Gadolinium Analysis

MRI studies of cell pellets were performed as previously described [21,26]. Briefly, BALB/c murine macrophage J774A.1 cells were cultured at 37°C with 5% CO_2_ in DMEM with 2 mM glutamine, 100 U ml^-1^ penicillin, 0.1 mg ml^-1^ streptomycin and 10% fetal bovine serum and grown to approximately 90% confluency in 25 cm^2^ tissue culture flasks. Cells were incubated for 2 h at 37°C using discoidal or spherical GBCA-HDL in complete DMEM at a concentration of 2 μM Rhodamine B. After incubation, cells were washed twice using PBS and collected into 15 ml BD Falcon tubes (BD Biosciences, San Jose, CA, USA). Cells were fixed using 200 μl of a 4% paraformaldehyde solution in PBS. The cells were dispersed and transferred into sealed 200 μl plastic pipette tips and allowed to settle overnight at 4°C to form loosely packed cell pellets. The pipette tips containing the cell pellets were transferred into a custom-made sample holder and 0.5 mM Magnevist (Bayer Healthcare, Wayne, NJ, USA) in saline (Baxter Healthcare, Deerfield, IL, USA) was added to the sample holder as a reference sample.

High resolution *T*_1_-weighted images were acquired using a custom-made solenoid transmit-receive coil on a Philips Achieva 3.0T X-series Quasar system with the following acquisition parameters: a spin echo sequence (repetition time/echo time, *TR/TE* = 600 ms/13 ms; FOV = 6.2 x 6.2 cm; matrix size = 308 x 308; slice thickness = 1.0 mm; number of averages = 4).

Images were analyzed using ImageJ (National Institutes of Health, Bethesda, MD, USA) to measure the intensities of the cell pellet after incubation with GBCA-HDL (*I*_treatment_), the cell pellet after incubation with medium (*I*_control_) and the standard deviation of the liquid outside of the cell pellet (*I*_noise_). The contrast to noise ratio (CNR), which is defined as CNR = (*I*_treatment_ − *I*_control_ *)*/*I*_noise_ was calculated. The normalized enhancement ratio (NER), which is defined as NER = [(*I*_treatment_ − *I*_control_ *)*/*I*_control_] x 100% was calculated.

### Mouse MRI Studies

All procedures were performed with guidelines and regulations for the use of vertebrate animals, including prior approval by the University of Massachusetts Medical School Animal Care and Use Committee. The procedures for ensuring that discomfort, distress, pain, and injury were limited to that which is unavoidable in the conduct of the proposed research and provided by the University of Massachusetts Medical School Animal Medicine Core Facility. Animal MRI studies were performed using male apo E knockout (KO) B6.129P2-*Apoe* ^*tm1Unc*^/J mice (Jackson Laboratory, Bar Harbor, ME, USA), which is a genetically engineered mouse model of atherosclerosis. Male wild type (WT) C57BL/6 J mice (Jackson Laboratory, Bar Harbor, ME, USA) were used as controls. Apo E KO mice (n = 9) and WT mice (n = 4) were kept on either a Western diet comprising of 21% fat and 0.15% cholesterol or a normal chow diet, respectively, for a period of at least 5 months. Sample size calculations were performed as previously described to determine the number of mice needed to detect a significant change [34]. MR imaging using a Philips Achieva 3.0T X-series Quasar system was performed before (pre-contrast imaging) and 4 h, 24 h, 48 h and 72 h after (post-contrast imaging) tail-vein administration of GBCA-HDL particles at a dose of 0.05 mmol Gd kg^-1^.

To set up anesthesia, a mobile laboratory animal anesthesia system was prepared (VetEquip, Livermore, CA, USA). Briefly, the isoflurane tank was filled to the top line using isoflurane (Piramal, Bethlehem, PA, USA). Mice were anesthetized using a 4% isoflurane-O_2_ gas mixture inside a vented 2L induction chamber (VetEquip, Livermore, CA, USA) fitted with an anesthesia gas filter unit (Bickford-Omnicon, Wales Center, NY, USA). An MRI reference sample comprising of 0.5 mM Magnevist in saline was affixed inside a custom-made solenoid transmit-receive coil. The anesthetized mouse was placed inside the solenoid transmit-receive coil and anesthesia was maintained by delivering a 1.5% isoflurane-O_2_ gas mixture through a nose cone. An in-house vacuum was turned on and the vacuum line was placed near the tail of the mouse to ensure that excess gases were removed from the area. The respiratory sensor was placed on the abdomen and connected to the small animal monitoring and gating system (SA instruments, Stony Brook, NY, USA) to monitor the respiratory rate. The isoflurane-O_2_ gas mixture was adjusted so that the respiratory rate was below 30 breaths per minute during image acquisition. A warming blanket (Stryker, Kalamazoo, MI, USA), which was connected to a warm water recirculator (Stryker, Kalamazoo, MI, USA) was used to keep the animal warm during the scan. After each MRI scan was completed, anesthetized mice were allowed to recover under a heat lamp. All mice recovered within 30 minutes with no adverse effects observed. Upon study completion, all animals were euthanized by CO_2_ asphyxiation.

High resolution *T*_1_-weighted multislice spin echo images were obtained using a custom-made solenoid transmit-receive coil on a Philips Achieva 3.0T X-series Quasar system using the following acquisition parameters: a spin echo sequence (*TR/TE* = 600 ms/13 ms; FOV = 3.0 x 3.0 cm; matrix size = 256 x 256; 22 contiguous 0.5 mm-thick aorta slices were obtained with slice 6 positioned at the right renal artery as a landmark; number of averages = 8; total scan time = 41 min). The slices were matched at each time point to the baseline pre-contrast scan. All mice provided analyzable data and were included in the final analysis.

Images were analyzed using ImageJ to measure intensity values in regions of interest containing tissue directly surrounding the vessel lumen in the aortic vessel wall (*I*_w_), the surrounding muscle (*I*_m_) and an area outside of the mouse (*I*_noise_). The CNR between the aortic wall and adjacent muscle was calculated using this formula: CNR = (*I*_w_ − *I*_m_)/*I*_noise_. The mean NER of the aortic wall relative to the surrounding muscle was calculated using this formula: NER = [(*I*_w_/*I*_m_)_post_ − (*I*_w_/*I*_m_)_pre_]/(*I*_w_/*I*_m_)_pre_ x 100%. Intensity values in regions of interest (*I*_ROI_) in the kidney and liver were also measured. The mean signal-to-noise ratio (SNR) for the regions of interest in the kidney and the liver were calculated using this formula: SNR = *I*_ROI_ / *I*_noise_. The formula to calculate the relative SNR increase is defined as: SNR increase = (SNR_post_ / SNR_pre_ − 1) x 100%.

Data are presented as the means ± standard deviation. Statistical computations were performed using GraphPad Prizm 6.0 (GraphPad, San Diego, CA, USA) analysis of variance (ANOVA). The ANOVA assumptions were ensured using the Kolmogorov-Smirnov test, and, where necessary, multiple comparisons by Bonferroni were performed. Results were considered statistically significant at *p* < 0.05.

## Results

### Paramagnetic and Fluorescent GBCA-HDL Complexes Are Uptaken by Macrophages *In Vitro*

**Fig 1:**
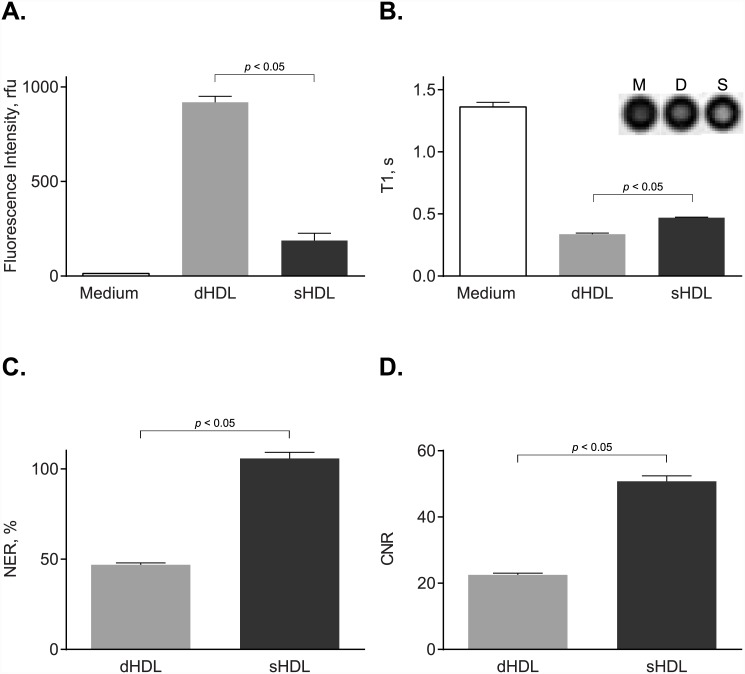
J774A.1 cell studies demonstrate uptake of paramagnetic and fluorescent HDL by macrophages *in vitro*. J774A.1 macrophages were incubated for 2 h at 37°C with medium only or with medium containing 2.0 μM (as calculated for Rhodamine B) paramagnetic and Rhodamine B-labeled discoidal HDL (dHDL) or spherical HDL (sHDL) synthesized using a 1:1 mixture of oxidized synthetic apo A-I peptides H4 and H6. (A) Fluorescence intensities of cell lysates were measured and normalized to total cell protein content (mean ± SD, n = 3). (B) Loosely packed cell pellets were generated and *T*_1_ values were measured using *T*_1_-weighted MR imaging. Shown in the inset are *T*_1_-weighted images of cell pellets incubated with medium alone (M), dHDL (D) or sHDL (S). (C) Normalized enhancement ratio (NER) values for cell pellets are calculated from corresponding *T*_1_-weighted images and are relative to cells incubated with medium only. (D) Contrast-to-noise ratio (CNR) values for cell pellets are calculated from the corresponding *T*_1_-weighted images and are relative to cells incubated with medium only.

To study *in vitro* macrophage uptake, we tested our paramagnetic and fluorescent HDL-like nanoparticles of either discoidal (dHDL) or spherical (sHDL) shape using the established J774A.1 macrophage line. To detect for Rhodamine B-labeled lipid uptake, fluorescent intensities in J774 cell lysates were measured after 2 h incubation at 37°C with either the dHDL or the sHDL formulations. Fig 1A shows the presence of Rhodamine B in the cell lysates of J774A.1 macrophages, indicating uptake of both dHDL and sHDL. To detect for Gd-labeled lipid uptake, *T*_1_-weighted MR imaging was performed on J774A.1 cell pellets after 2 h incubation at 37°C with either the dHDL or sHDL formulations. Fig 1B shows that cells incubated with either dHDL or sHDL have short T1 values, indicating the presence of Gd uptaken by the cell. Accordingly, dHDL and sHDL have mean NER values of 47 and 105, respectively, and CNR values of 23 and 50, respectively (Fig 1C and 1D). Together, these results indicate that both the paramagnetic and the fluorescent components of the dHDL and sHDL formulations are delivered to J774A.1 macrophages *in vitro*.

### Detection of GBCA-HDL Complexes in the Cytoplasm of Macrophages *In Vitro* Demonstrates Targeted Intracellular Delivery

**Fig 2:**
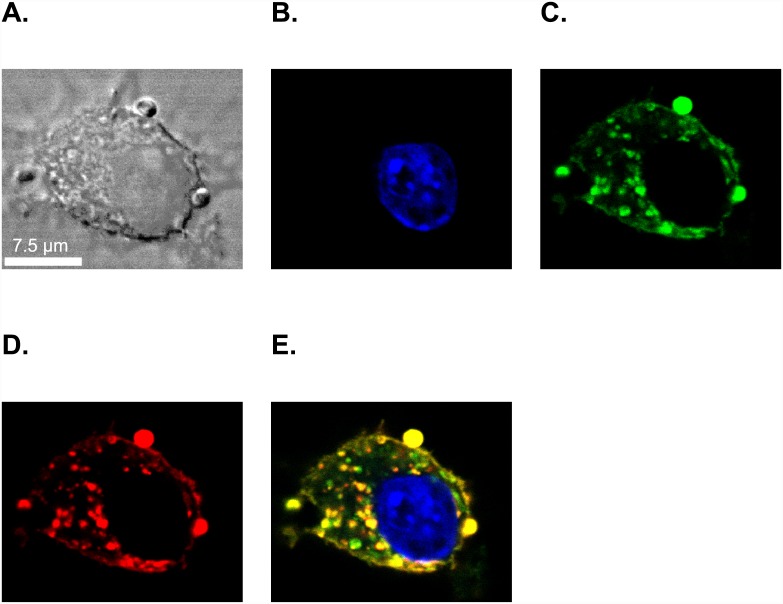
Confocal microscopy confirms the targeted delivery of both lipid and peptide components of GBCA-HDL to the cytoplasm of J774A.1 macrophages. J774A.1 cells were incubated for 2 h at 37°C with medium only or with medium containing 2.0 μM (as calculated for Rhodamine B) paramagnetic and Rhodamine B-labeled discoidal HDL (dHDL) or spherical HDL (sHDL) synthesized using a 1:1 mixture of oxidized synthetic apo A-I peptides H4 and H6. A small portion of the peptide H4 in the dHDL and sHDL preparations was fluorescently labeled with Dylight 488. (A) Differential interference contrast (DIC) image of a single representative J774A.1 macrophage incubated with sHDL (similar images were obtained for dHDL). The cell was stained with 4’,6-diamino-2-phenylindole (DAPI) dye (blue, B), visualized for Dylight 488-labeled peptide H4 (green, C) and Rhodamine B-labeled lipid (red, D). (E) The merged image shows colocalization of Dylight 488-labeled peptide H4 with Rhodamine B-labeled lipid in the cytoplasm of J774A.1 macrophages, indicating specific uptake of intact sHDL particles into the cytoplasm. White scale bar = 7.5 μM.

To determine that the paramagnetic and fluorescent dHDL and sHDL particles are specifically uptaken by J774A.1 macrophages and not a result of lipid exchange [35], we labeled the oxidized synthetic apo A-I H4 peptide using Dylight 488. We hypothesized that if uptake is specific, the Dylight 488-labeled oxidized Apo-AI H4 peptide along with the Rhodamine B-labeled lipid would be observed inside the cell. J774A.1 macrophages were incubated with the paramagnetic and fluorescent dHDL and sHDL formulations for 2 h at 37°C and the cells were fixed and visualized by confocal microscopy. The images in Fig 2 (shown for sHDL; similar results were obtained for dHDL) demonstrate the presence of Rhodamine B-labeled lipid (Fig 2D) and Dylight 488-labeled oxidized H4 peptide (Fig 2C) inside the cytoplasm, which clearly indicates that GBCA-HDL were specifically uptaken by J774A.1 macrophages. Pronounced colocalization of both fluorescently labeled lipid and peptide molecules shown on the merged image in Fig 2E also demonstrates that at this time point most of the GBCA-HDL particles are intact.

### Paramagnetic and Fluorescent HDL Complexes Deliver the Incorporated Contrast Agents into Macrophage-Rich Atherosclerotic Plaques *In Vivo*

**Fig 3:**
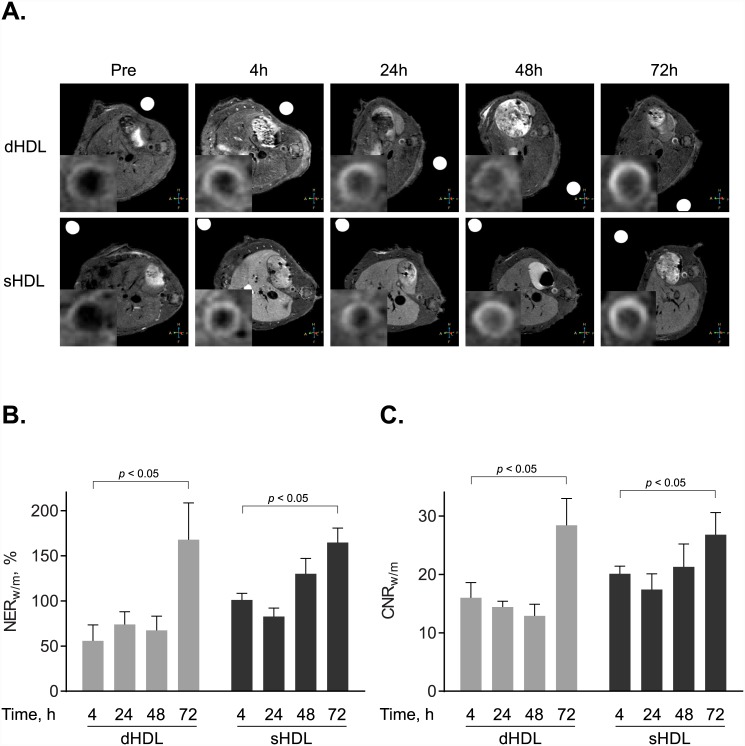
Targeted *in vivo* imaging of macrophages in aortic atherosclerotic lesions. (A) Representative axial *T*_1_-weighted images of apo E knockout (KO) mice, collected before (Pre) and 4 h, 24 h, 48 h and 72 h after contrast agent administration. Apo E KO mice were administered with the equivalent of 0.05 mmol Gd kg ^−1^ of paramagnetic and Rhodamine B-labeled discoidal HDL (dHDL) or spherical HDL (sHDL) containing a 1:1 mixture of oxidized synthetic apo A-I peptides H4 and H6. The insets in each scan show the original images, which were cropped and enlarged to highlight the aorta. A single representative mouse is shown for either dHDL or sHDL. (B) NER values (mean ± SD) of mouse aorta wall following contrast administration were calculated for each of the time points (n = 5 slices) for the representative mouse using the corresponding *T*_1_-weighted images and are relative to muscle. (C) CNR values (mean ± SD) of mouse aorta wall following contrast administration were calculated for each of the time points (n = 5 slices) using the corresponding *T*_1_-weighted images and are relative to muscle.

To evaluate if paramagnetic and fluorescent dHDL and sHDL particles target atherosclerotic plaques *in vivo*, we utilized apo E KO mice, which are known to develop plaques when fed a high fat, high cholesterol Western diet. For controls, age-matched WT mice fed a normal chow diet were utilized. After 5 months on the Western diet or a normal chow diet, apo E KO mice (n = 9) and WT mice (n = 4), respectively, were injected intravenously with a single dose of the paramagnetic and fluorescent dHDL or sHDL formulations at a dose of 0.05 mmol Gd kg ^−1^. Axial *T*_1_-weighted images of the abdominal aorta were taken before (pre-imaging) and 4h, 24h, 48h and 72h after contrast agent administration. Fig 3A shows representative images of aorta sections from two apo E KO mice, injected with either dHDL or sHDL. The NER (Fig 3B) and CNR values (Fig 3C) were calculated relative to muscle. The mean NER values for dHDL and sHDL at 72 h were 168% and 165%, respectively. The mean CNR values for dHDL and sHDL at 72 h were 28 and 27, respectively. Combined, the results clearly demonstrate that the generated GBCA-HDL formulations can be used for diagnostic imaging of atherosclerotic plaques *in vivo*. The data also indicate that the NER and CNR values increase with time and reach a maximum at 72 h, which increases the diagnostic power of the method. Importantly, WT mice showed no contrast enhancement (results not shown), consistent with results from our previous study [26].

### Organ Clearance of GBCA-HDL Depends on Particle Shape and Composition

**Fig 4:**
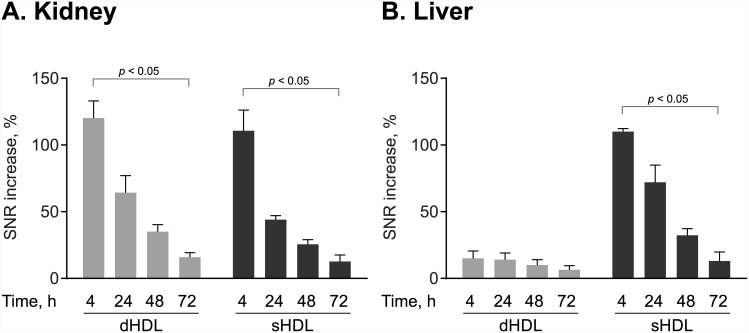
The organ clearance depends on particle shape and composition. The relative signal-to-noise-ratio (SNR) increase values were calculated for regions of interest in the kidney (A) and the liver (B) of apo E KO mice at 4 h, 24 h, 48 h, 72 h following injection of the equivalent of 0.05 mmol Gd kg ^−1^ of paramagnetic and Rhodamine B-labeled discoidal HDL (dHDL) or spherical HDL (sHDL) containing a 1:1 mixture of oxidized synthetic apo A-I peptides H4 and H6. The data were calculated for each of the timepoints for a representative mouse receiving either dHDL or sHDL using the corresponding *T*_1_-weighted images and are represented as percent SNR increase relative to the baseline pre-contrast scan (mean ± SD). Statistical difference between the 4 h and 72 h time points is shown.

To preliminarily evaluate how GBCA-HDL are excreted, the SNR values of the kidney and liver were measured before and after contrast administration as previously described [36]. Fig 4A shows that in the kidney for mice receiving both dHDL and sHDL, the relative SNR increase values quickly decrease with time from 120% at 4 h to 16% at 72 h for discoidal GBCA-HDL and from 111% at 4 h to 12% at 72 h for spherical GBCA-HDL. In the liver, the relative SNR increase values were negligible for dHDL at all time points after contrast administration, indicating that dHDL particles might be largely excreted through the kidney. In contrast, the liver SNR values observed for sHDL (Fig 4B) demonstrate hepatic clearance for these particles. These data clearly indicate that the organ clearance of GBCA-HDL depends on particle shape and composition.

## Discussion

Discriminating vulnerable plaques from stable plaques is of paramount importance. One of the challenging aspects in distinguishing between the two is choosing a distinctive target that is mostly present in vulnerable plaques [14]. In this regard, we target macrophages as their presence positively correlates with plaque vulnerability [9,10]. By employing a naturally occurring oxidative modification in the apo A-I protein or synthetic apo A-I peptides, which allows for the physiological delivery of GBCA-HDL to macrophages in atherosclerotic plaques, we further avoid any potential side effects that might surface with the use of targeting moieties. Importantly, we have achieved higher calculated CNR and NER for the aortic wall relative to muscle using this method as compared to other studies employing targeting moieties to macrophages [21,24]. This clearly demonstrates the effectiveness of our method. It’s important to note that our high-resolution MRI results were obtained using a custom-made solenoid transmit-receive coil and a 3.0T clinical scanner, which is nearly three-fold lower in magnetic field strength as compared to scanners routinely utilized in small animal MRI [21,24].

Another important issue is related to Gd toxicity as GBCAs have been associated with the serious and sometimes fatal condition known as NSF [16,18,37]. Importantly, a positive correlation between GBCA dose and increased side effects has been shown [18]. Currently, most commercially available contrast agents are largely excreted through the kidney, which is believed to contribute to the onset of NSF, particularly in patients with impaired renal function [37]. Therefore, reducing the risk of NSF associated with the use of GBCAs is of great clinical importance. Two proposed methods to reduce the risk of NSF are: 1) minimizing the administered GBCA dose and 2) diverting GBCA clearance from the kidney to the liver [37].

To help reduce the risk of NSF and increase the diagnostic power of our method as compared to our previous study [26], we increased the Gd-content per particle, while maintaining the same total amount of GBCA-HDL injected at a dose of 0.05 mmol Gd kg^-1^. This as well as targeted delivery of our GBCA-HDL to macrophages demonstrated both *in vitro* (Figs 1 and 2) and *in vivo* (Fig 3; see also [26]) not only minimized the undesired exposure of GBCAs but also resulted in significantly increased contrast enhancement in the atherosclerotic aorta wall (Fig 3B and 3C). Additionally, in this study, we have evaluated the organ clearance of our discoidal and spherical GBCA-HDL by measuring the SNR values in regions of interest in the kidney and liver and found that in contrast to discoidal GBCA-HDL that are largely excreted through the kidney (Fig 4A), spherical GBCA-HDL exhibit hepatic clearance (Fig 4B). The observed hepatic clearance of spherical GBCA-HDL may reduce the risk of NSF in patients. In addition, our findings demonstrate that spherical GBCA-HDL can be used for liver MRI applications as well.

The results obtained using the proposed contrast-enhanced imaging technology for the detection of vulnerable atherosclerotic plaques strongly encourage further development of our method for diagnostic imaging of atherosclerosis. In addition, broader applications in other inflammation-based disorders where macrophages are known to play a key role, such as rheumatoid arthritis and cancer might be readily achieved and are currently under investigation.

## Conclusions

Magnetic resonance imaging of macrophage-enriched vulnerable atherosclerotic plaques is achieved by employing lipopeptide nanoparticles that contain gadolinium-based contrast agents and modified synthetic apolipoprotein A-I peptides, which target the particles to macrophages *in vitro* and *in vivo*. This is a novel methodology that could substantially improve early diagnosis and prognosis of atherosclerosis as well as treatment efficacy evaluation.
